# Molecular and cellular effects of a novel hydroxamate-based HDAC inhibitor – belinostat – in glioblastoma cell lines: a preliminary report

**DOI:** 10.1007/s10637-016-0372-5

**Published:** 2016-07-29

**Authors:** Magdalena Kusaczuk, Rafał Krętowski, Anna Stypułkowska, Marzanna Cechowska-Pasko

**Affiliations:** Department of Pharmaceutical Biochemistry, Medical University of Białystok, Mickiewicza 2A, 15-222 Białystok, Poland

**Keywords:** Apoptosis, Belinostat, Glioblastoma, Glucose regulated proteins, Histone deacetylase inhibitors

## Abstract

Histone deacetylase (HDAC) inhibitors are now intensively investigated as potential cytostatic agents in many malignancies. Here, we provide novel information concerning the influence of belinostat (Bel), a hydroxamate-based pan-HDAC inhibitor, on glioblastoma LN-229 and LN-18 cells. We found that LN-229 cells stimulated with 2 μmol/L of Bel for 48 h resulted in 70 % apoptosis, while equivalent treatment of LN-18 cells resulted in only 28 % apoptosis. In LN-229 cells this effect was followed by up-regulation of pro-apoptotic genes including *Puma*, *Bim*, *Chop* and *p21*. In treated LN-18 cells only *p21* was markedly overexpressed. Simultaneously, LN-229 cells treated with 2 μmol/L of Bel for 48 h exhibited down-regulation of molecular chaperones GRP78 and GRP94 at the protein level. In contrast, in LN-18 cells Western blot analysis did not show any marked changes in GRP78 nor GRP94 expression. Despite noticeable overexpression of *p21*, there were no signs of evident G1 nor G2/M cell cycle arrest, however, the reduction in number of the S phase cells was observed in both cell lines. These results collectively suggest that Bel can be considered as potential anti-glioblastoma agent. To our knowledge this is the first report presenting the effects of belinostat treatment in glioblastoma cell lines.

## Introduction

Glioblastoma is considered one of the most abundant type of glial tumors with poor prognosis of survival. Surgical resection followed by chemotherapy combined with radiotherapy is now one of the prime adjuvant treatments available for glioblastoma. Although extensive efforts to treat this cancer are currently being explored, there are still no strategies effective enough to cure this malignancy or efficiently improve patient outcomes. The main obstacles to overcome in order to develop effective therapies against glioblastoma include high heterogeneity and anaplasticity of these cancer cells together with the high potential for migration into the brain. Thus, new modalities of anticancer drugs are required to advance the treatment of malignant gliomas [1, 2].

Recent findings confirm that cancer development is regulated on both genetic as well as epigenetic levels. Genetic alterations in malignant gliomas have been thoroughly studied and are known to include mutations in the main tumor suppressor genes such as *p53* or *Pten* [3] and deletions of some parts of the chromosomes (e.g. 1p36.23, 6q26–27, 17p13.3–12) [4]. Recently, there has been growing body of evidence to suggest epigenetic regulation affects cancerogenesis and cancer progression. Methylation of the CpG islands in gene promoters and remodeling of the chromatin structure have also been identified as important mechanisms involved in oncogenesis [5]. Modifications of the chromatin architecture may be regulated by histone acetylation and deacetylation [5]. Nucleosomes composed of sparsely acetylated histones are the hallmark of transcriptionally silent chromatin, whereas the relaxed chromatin structure is characterized by densely acetylated histone proteins [5, 6]. The two crucial groups of counterworking enzymes responsible for guarding histone acetylation status are histone acetyltransferases (HATs) and histone deacetylases (HDACs). HATs are responsible for transferring acetyl moieties from acetyl-coenzyme A onto the amino groups of lysine residues of histones, which induces transcription. In opposition, HDACs remove these acetyl groups from histone proteins, resulting in chromatin condensation and suppression of transcriptional activity [5, 6]. Importantly, a growing number of studies identifying non-histone protein acetylation are being published [7–9]. The list of non-histone proteins known to be acetylation targets is constantly expanding and it includes essential cellular signaling mediators and transcription factors [9, 10].

Moreover, the latest reports suggest that molecular chaperones might also be the substrates of posttranslational modification through protein acetylation [7, 8, 11]. It has been shown that HDAC6 is capable of regulating endoplasmic reticulum (ER) stress status via alterations in the acetylation level of heat-shock protein 90 (HSP90) [8]. Another ER chaperone being investigated in the context of acetylation-dependent regulation is glucose-regulated protein 78 (GRP78), which is known to be a central regulatory molecule in the unfolded protein response (UPR). The GRP78 has recently been demonstrated to be acetylated following HDAC inhibition resulting in UPR activation [11, 12]. These results are particularly relevant since overexpression of GRP78, together with the other ER-resident molecular chaperone GRP94, has been associated with a number of malignant tumors and seems to be of critical importance in glioblastoma biology [13, 14]. These findings suggest an acetylation-dependent model of regulation that extends beyond the chromatin level.

Acetylation homeostasis may be modified by the group of pharmacologically potent compounds called the histone deacetylase inhibitors (HDACIs). Bel is a novel hydroxamate-based inhibitor of class I and class II HDACs demonstrating in vitro activity against a variety of human cell lines and in vivo activity against bladder, ovarian, and colon cancer xenografts [15–17]. Recently, Bel has also been evaluated in clinical trials in patients with hematological malignancies [18, 19] and solid tumors [20, 21]. Even though considerable research concerning Bel function in cancer has already been undertaken, the mechanisms of cellular responses and gene expression patterns initiated after Bel treatment are not universal and seem to be specific to cell type. Given this research, the mode of action of Bel in cancer cells has been attributed to reduced proliferation [22–24], increased apoptosis [23–25], and cell cycle arrest [24]. However, the molecular pathways underlying these processes have not been resolved.

Although favorable antineoplastic effects of belinostat have been demonstrated in various models of malignancies, brain tumors are still an unexplored area of investigation. Thus, modulating HDAC activity in brain tumors requires further research in anticancer therapy. This study was designed to evaluate the effect of Bel on proliferation and apoptosis of glioblastoma LN-229 and LN-18 cells. Since there are no studies reporting Bel efficiency in brain tumors, we investigated its use as a potential epigenetic-based cytostatic agent for treatment of glioblastomas.

This research demonstrated that Bel inhibited growth in both LN-229 and LN-18 cell lines. Results indicate that LN-229 as well as LN-18 cells showed significant dose- and time-dependent inhibition of cell proliferation. Although there was no clear evidence of G1 nor G2/M cell cycle arrest, the cell cycle was visibly disrupted with the reduction of the S phase cells in both tested cell lines. However, we found a prominent induction of apoptotic cell death occurred in LN-229 cells exposed to 48-h treatment with 2 μmol/L of Bel. In contrast, LN-18 cells appeared to be more resistant to Bel-mediated apoptosis. Additionally, we analyzed the expression of *Puma*, *Noxa*, *Bax*, *Bim*, *Bcl-2*, *Bcl-X*_*L*_, *p21* and *Chop* in both cell lines after treatment with Bel. The pro-apoptotic *Puma*, *Bim* and *Chop* were significantly up-regulated in Bel-treated LN-229 cells, and *p21* was significantly overexpressed in both cell lines. In addition, the molecular chaperones GRP78 and GRP94 were significantly down-regulated at the protein level in LN-229 cells. In contrast, Western blot analysis did not reveal any significant difference in chaperone expression in LN-18 cells. These results suggest that LN-18 cells are more Bel-resistant due to elevated expression of cytoprotective molecules and lack of concomitant up-regulation of pro-apoptotic factors. This further suggests that Bel is a candidate drug for anti-glioblastoma therapy. Further research is still necessary to comprehensively determine markers of Bel-sensitivity to overcome the limitations of its usefulness as a potential cytostatic agent in this type of cancer.

## Materials and methods

### Reagents

Dulbecco’s modified Eagle’s medium (DMEM), containing glucose at 4.5 mg/mL (25 mmol/L) with Glutamax, penicillin, streptomycin, and trypsin-EDTA were provided by Invitrogen (San Diego, USA). High Capacity RNA-to-cDNA Kit was purchased from Applied Biosystems (Foster City, CA). ReliaPrep RNA Cell Miniprep System, and HDAC-Glo ™ I/II Assay and Screening System were provided by Promega (Madison, USA); FBS Gold by Gibco (San Diego,USA); fluorescein isothiocyanate (FITC) Annexin V Apoptosis Detection Kit I by BD Pharmingen (CA, USA); RIPA lysis buffer and BCA Protein Assay Kit by Thermo Scientific (Rockford, USA); RNase by AppliChem (Darmstadt, Germany). Sigma-Fast BCIP/NBT reagent and molecular-grade purity water were provided by Sigma (St. Louis, MO, USA). The polyclonal (mouse) anti-KDEL antibody was purchased from Enzo Life Sciences, Inc. (Lausen, Switzerland). Alkaline phosphatase-conjugated anti-mouse immunoglobulin G (IgG) was from Rockland (Pennsylvania, USA). Alkaline phosphatase-conjugated anti-rabbit immunoglobulin G and polyclonal (rabbit) anti-β-tubulin antibody were provided by (Cell signaling (Boston, USA). Belinostat was purchased from MedChem Express (Stockholm, Sweden).

### Cell cultures

Human glioblastoma cell lines LN-229 and LN-18 were a kind gift of Prof. Cezary Marcinkiewicz from the Department of Neuroscience, Temple University, Philadelphia, USA. Cells were cultured in high-glucose DMEM with addition of 5 % of heat-inactivated fetal bovine serum GOLD (FBS GOLD), streptomycin (100 μg/mL), penicillin (100 U/mL), and 2 mmol/L L-glutamine. Cells were cultivated in Falcon flasks (BD) in a 5 % CO_2_ incubator (Galaxy S+; New Brunswick) at the temperature of 37 °C. Cells reaching sub-confluency were detached from the culture dishes using 0.05 % trypsin 0.02 % EDTA in calcium-free phosphate-buffered saline (PBS) and counted in cell counter Scepter (Millipore). Belinostat was dissolved in dimethyl sulfoxide (DMSO) as 50 mmol/L stock solution and subsequently diluted in growth media keeping the final concentration of DMSO ≤1 % in culture.

### Determination of HDAC inhibitor potency

The measurement of HDAC activity after Bel treatment was performed using luminescent HDAC-Glo ™ I/II Assay and Screening System (Promega) following the manufacturer’s specifications. In brief, cells at a density of 10,000 cells/well were seeded in a white-walled 96-well culture plate (Nunclone). Cells were allowed to attach and then incubated with medium containing Bel in concentrations ranging from 0.1–10 μmol/L at 37 °C for 1 h. After incubation, 100 μL of the HDAC-Glo ™ I/II Reagent with Triton® X-100 at a final concentration of 1 % was added to each well, and cells were left in the dark at room temperature for additional 35 min. Luminescence was read using a microplate reader (Tecan, Switzerland) at a signal steady-state 35 min after adding the HDAC-Glo ™ I/II Reagent to the cells. The experiment was run in triplicate.

### Cell viability

The viability of the cells was evaluated according to the method of Carmichael et al. [26] using 3-(4,5-dimethylthiazol-2-yl)-2,5-diphenyltetrazolium bromide (MTT). In brief, cells were seeded in 24-well plates at a density of 5 × 10^4^ per well. Confluent cells were exposed to various concentrations of Bel (0.1–10 μmol/L) for 24 and 48 h. Then, the cells were washed twice with PBS and incubated with 1 mL of MTT solution (0.25 mg/mL in PBS) at 37 °C in a humidified 5 % CO_2_ atmosphere for 4 h. The medium was removed and formazan products were solubilized in 1 mL of 0.1 mmol/L HCl in absolute isopropanol. The absorbance of a converted dye in living cells was read on a microplate reader (Tecan) at λ = 570 nm. The viability of Bel-treated LN-229 and LN-18 cells was calculated as a percentage of control non-treated cells. All experiments were run in triplicate.

### Cell morphological analysis

To visualize morphological specificity of the glioblastoma, LN-229 and LN-18 cells exposed to Bel treatment and then stained with acridine orange (AO)/ethidium bromide (EtB). Staining was followed by fluorescent and phase contrast microscopic observation. Acridine orange enters both viable as well as dead cells. AO emits green fluorescence when it is bound to double-stranded DNA in viable cells, and red fluorescence, when it is bound to single-stranded DNA observed predominantly in dead cells. EtB is thrown out from living cells [27]. The LN-229 and LN-18 cells, at a density of 2.5 × 10^5^, were seeded into 6-well plates and incubated with 0.5 or 2 μmol/L of Bel at 37 °C in a humidified atmosphere containing 5 % CO_2_ for 48 h. After incubation, the cells were stained with a mixture of AO (10 μmol/L) and EtB (10 μmol/L). The cells were visualized using a fluorescent and phase contrast microscope (Olympus, CKX 41) at 100× or 200× magnification.

### Detection of apoptosis

Apoptotic LN-229 and LN-18 cells were detected using FITC Annexin V apoptosis detection Kit I followed by flow cytometry analysis. The cells were seeded in a 6-well plate at a density of 2.5 × 10^5^ per well (in 2 mL of medium) and cultivated until they were confluent. The cells were maintained in high-glucose DMEM with 0.5 or 2 μmol/L of Bel for 24 and 48 h. Next, cells were detached by trypsinization and re-suspended in DMEM and subsequently a binding buffer. The cells were assayed using FITC Annexin V and propidium iodide (PI) staining and protected from light exposure for 15 min according to the manufacturer’s manual. The FACSCanto II cytometer (Becton Dickinson) was used to perform flow cytometry analysis. The FACSDiva software was used for data analysis. The dead cells were discriminated on the basis of forward- and side-scatter parameters; Annexin V+/PI- were identified as early apoptotic and Annexin V+/PI+ as late apoptotic cells. A sum of Q2 and Q4 quadrant populations of analyzed cells was presented as the percentage of apoptotic cells.

### Cell cycle analysis

The distribution of the cell cycle phases was analyzed by flow cytometry. Briefly, LN-229 and LN-18 cells were seeded into 6-well plates at a density of 2.5 × 10^5^ cells per well and treated with 0.5 or 2 μmol/L of Bel for 24 and 48 h. After incubation, the cells were harvested and then fixed with 1 mL of 70 % ethanol and kept overnight at −20 °C. Before analysis, the cells were re-suspended in PBS, treated with 50 μg/mL of DNase-free RNase A (AppliChem), and stained with 100 μg/mL of PI. The FACSCanto II flow cytometer was used to read the fluorescence.

### RNA isolation

Total RNA was isolated using ReliaPrep RNA Cell Miniprep System (Promega) with DNase I treatment according to the manufacturer’s instructions. Spectrophotometric measurements were done to assess the quantity of the extracted RNA (NanoPhotometer, Implen, Germany). In order to evaluate the quality of the isolated RNA, RIN (RNA integrity number) values were determined by capillary electrophoresis using a Bioanalyzer 2100 (Agilent, Palo Alto, CA). High-quality RNA samples (RIN > 9) were retained for further analyses.

### Gene expression analysis

Synthesis of the cDNA was performed using High Capacity RNA-to-cDNA Kit (Invitrogen) following the manufacturer’s instructions. In brief, 1 microgram of purified total RNA was used in a 20-μL reaction mixture containing random octamers, oligo dT-16 primers, dNTPs and MuLV Reverse transcriptase (RT). 2 μL of cDNA was used as a template for real-time qPCR reactions. Product amplification was performed using 2xHS-PCR Master Mix SYBR A (A&A Biotechnology, Poland). Primer sequences for *p21*, *Bax*, *Bim*, *Bcl-2*, *Bcl-X*_*L*_ were previously described [10]. The sequences of the other PCR primers were as previously described: *Puma*: F-GGAGCAGCACCTGGAGTC, R-TACTGTGCGTTGAGGTCGTC [28], *Noxa*: F-AGC TGGAAGTCGAGTGTGCT, R-ACGTGCACCTCCTGAGAAAA [28], *Chop*: F-CAGAACCAGCAGAGGTCACA, R-AGCTGTGCCACTTTCCTTTC [29], and for housekeeping *RPL13A*: F-CTATGACCAATAGGAAGAGCAACC, R-GCAGAGTATATGACCAGGTGGAA [10]. Additional assessment of primer accuracy was done using Primer-BLAST software. The following reaction parameters were applied: initial denaturation at 95 °C for 3 min, followed by 40 cycles of 95 °C for 1 min, 60 to 69 °C for 30 s, and 72 °C for 45 s. The CFX Connect Real-Time PCR System (Bio-Rad) was used to perform a real-time qPCR assay. Reactions were run in triplicates and expression was analyzed using the relative quantification method modified by Pfaffl [30].

### Protein assay

After treatment, cells were washed with cold PBS and solubilized in 100 μL of RIPA lysis buffer per well. The lysed cells were then subjected to centrifugation (12,000×g at 4 °C for 10 min), and supernatants were collected for protein assessment. The BCA Protein Assay Kit (Thermo Scientific, USA) was used to determine protein concentration in cell lysates. The method of Smith et al. [31] was applied for protein assay. Bovine serum albumin was used as a standard.

### Sodium dodecyl sulfate/polyacrylamide gel electrophoresis (SDS/PAGE)

Samples of the lysates containing 20 μg of protein were subjected to SDS–PAGE electrophoresis, as described by Laemmli [32]. Electrophoresis was run for 40–45 min using a 7.5 % polyacrylamide gel and constant current of 25 mA was applied.

### Immunoblotting

The resolved proteins were transferred to nitrocellulose membranes and pre-incubated with Tris-buffered saline (TBS) containing 0.05 % Tween 20 (TBS-T) and 5 % non-fat dry milk for 2 h. Membranes were soaked in a mixture of polyclonal (mouse) anti-KDEL antibody (1:1000) or polyclonal (rabbit) anti-β-tubulin antibody (1:1000) in 5 % dried milk in TBS-T at 4 °C for 16 h. Next, a 1-h incubation with secondary alkaline phosphatase-conjugated antibody against mouse or rabbit IgG at the 1:2500 dilution was carried out. Finally, the nitrocellulose membranes were washed five times with TBS-T and exposed to Sigma-Fast BCIP/NBT reagent.

### Statistical analysis

Results are presented as means ± SD from three independent experiments run in triplicate. Statistica Data Miner (StatSoft, Poland) was used to perform statistical analyses. A one-way analysis of variance (ANOVA) was carried out for comparisons between control and treated groups. Pair-wise comparisons were made by the post-hoc Tukey test. To count the half-maximal inhibitory concentration (IC_50_) values the GraphPad Prism 5 software (GraphPad Software, Inc., USA) was used. The differences were considered significant at *p* < 0.05.

## Results

### The effect of bel on HDAC activity

**Fig. 1:**
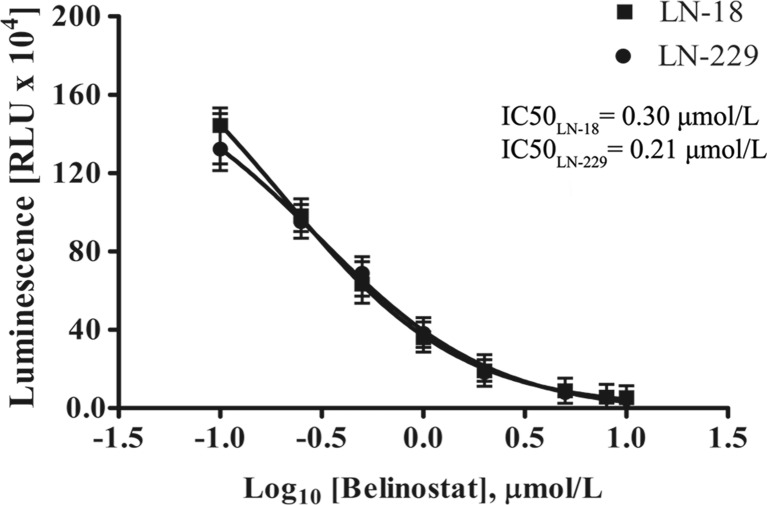
HDAC inhibitor potency of belinostat. Data were plotted against logarithmic values of Bel concentrations (ranging from 0.1 to 10 μmol/L), and IC_50_ values of HDAC inhibition were evaluated using GraphPad Prism 5 software. Data represent the mean ± standard deviations of three replicates

A luminescence-based HDAC-Glo ™ I/II Assay and Screening System (Promega) was used to confirm the HDAC inhibitory effect of Bel in LN-229 and LN-18 cells. The relative activity of class I and class II HDAC enzymes was evaluated in cells exposed to Bel in concentrations ranging from 0.1 to 10 μmol/L. It has been recognized that Bel effectively suppressed the activity of HDACs in both glioblastoma cell lines. The inhibitory effect was clearly dose-dependent (Fig. 1). Next, luminescence measurements were plotted against logarithmic values of equivalent Bel concentrations and the GraphPad Prism 5 software was used to calculate IC_50_ values of HDAC inhibition. The estimated IC_50_ values were 0.21 μmol/L for LN-229 and 0.30 μmol/L for LN-18 cells, however, these differences were not of statistical relevance.

### The effect of bel on cell viability

**Fig. 2:**
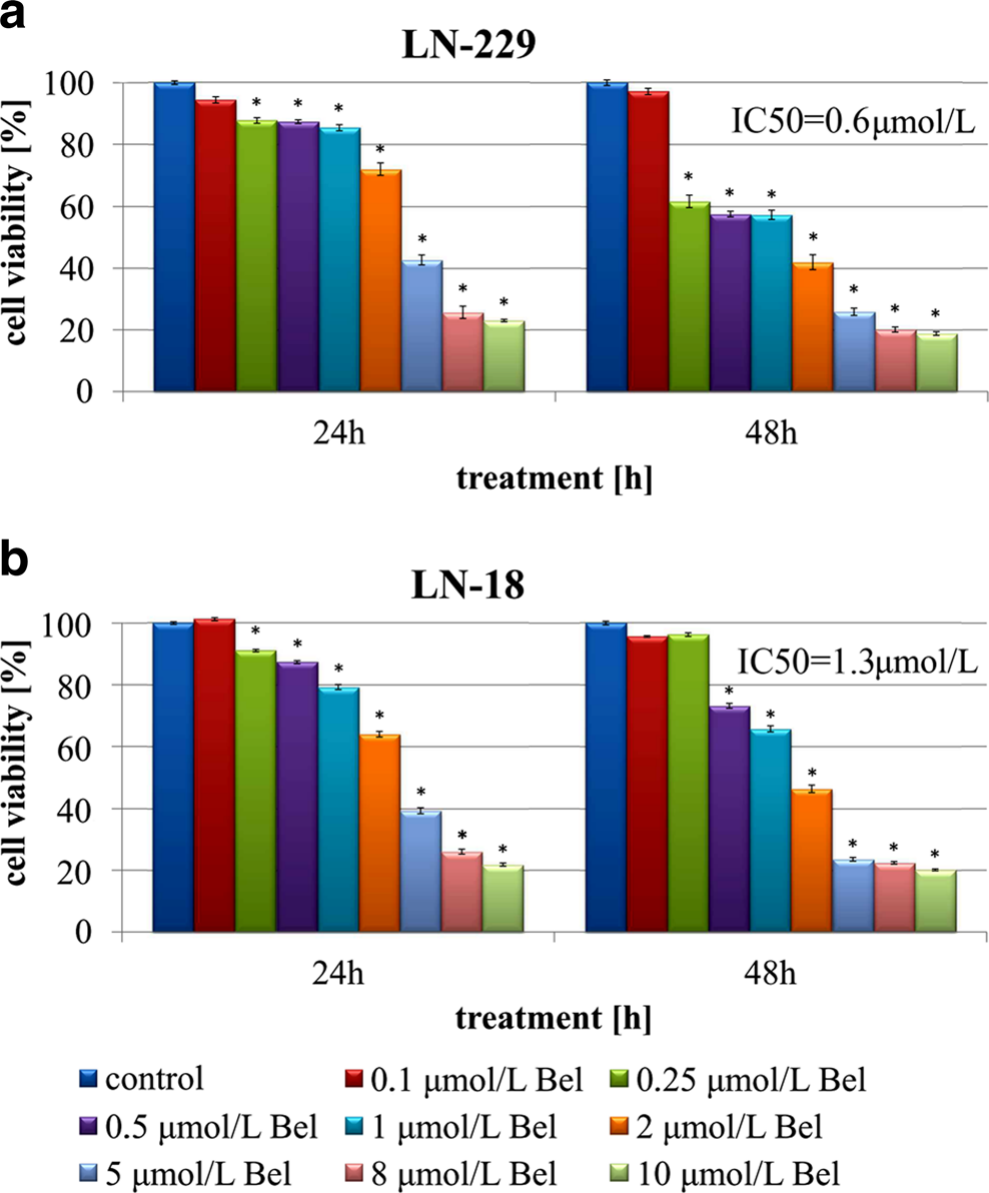
The viability of glioblastoma LN-229 (**a**) and LN-18 (**b**) cell lines treated with different concentrations of belinostat for 24 and 48 h. The results represent means for pooled triplicate values from three independent experiments. Significant changes are expressed relative to controls and marked with asterisks. Statistical significance was considered if * *p* < 0.05

The MTT method was used to assess the anti-proliferative effect of Bel on LN-229 and LN-18 glioblastoma cell lines. Initially, cells were exposed to increasing concentrations of Bel for 24 and 48 h. Belinostat exposure at concentrations ranging from 0.1 to 10 μmol/L caused dose-dependent and time-dependent reduction of LN-229 cell viability (Fig. 2a). In LN-18 cells the anti-proliferative effect of Bel was clearly dose-dependent, but seemed to be time-dependent to a lesser extent (Fig. 2b). The viability of both glioblastoma cell lines was inhibited as soon as 24 h after exposure to Bel. In comparison to cells exposed to lower concentrations of Bel, cells treated with 10 μmol/L of Bel showed the most pronounced effect on cell viability, approaching nearly 80 % of total inviable cells in both cell lines (Fig. 2). The GraphPad Prism 5 software was used to calculate the IC_50_ values of Bel activity. The LN-229 and LN-18 cells showed significantly different IC_50_ values of approximately 0.6 μmol/L and 1.3 μmol/L, respectively. Given this result, two representative concentrations of Bel at lower (0.5 μmol/L) and higher (2 μmol/L) than estimated IC_50_ values were selected for further examination.

### The effect of bel on cell morphology

**Fig. 3:**
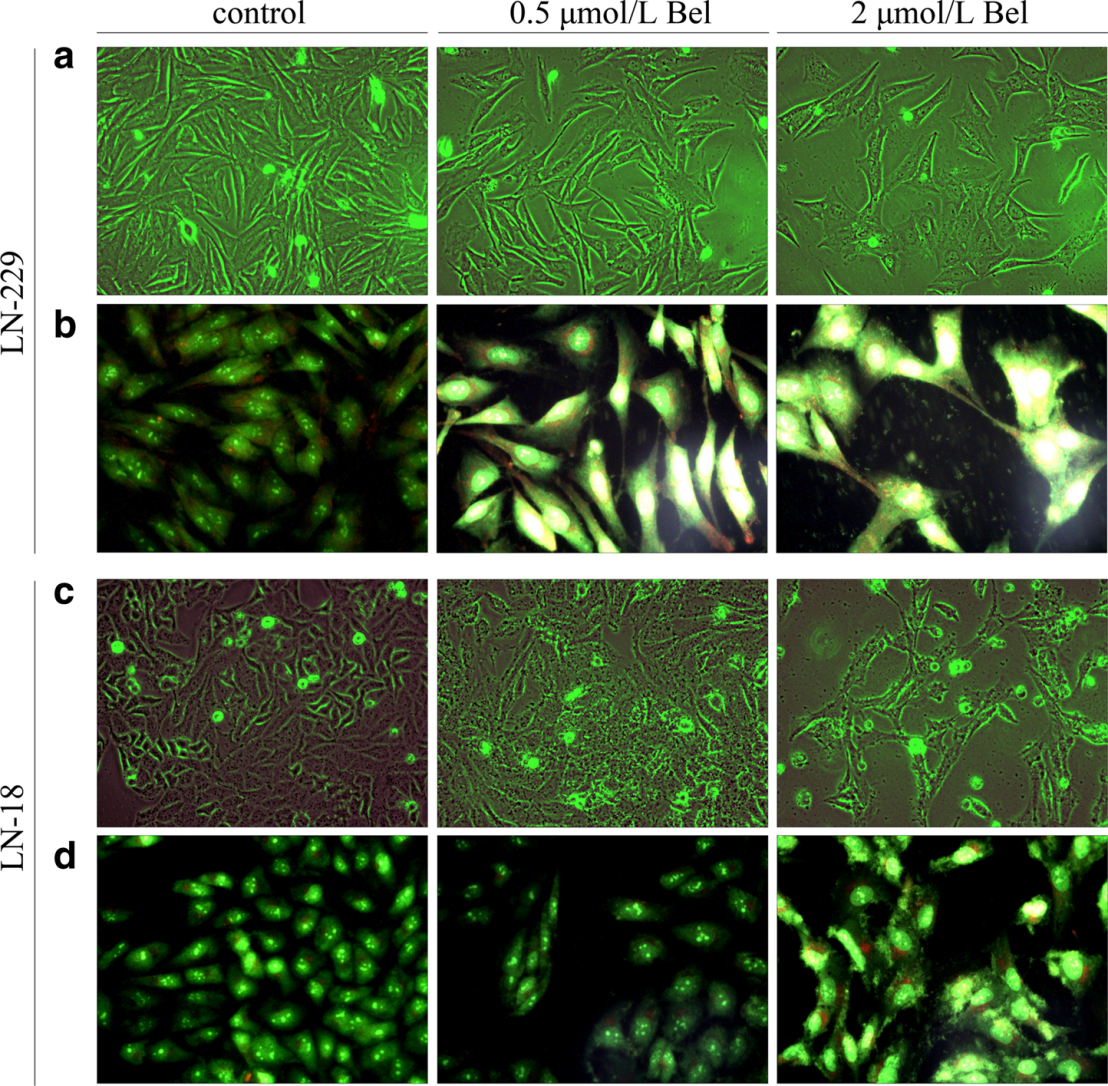
The phenotypic characteristics of LN-229 and LN-18 glioblastoma cells after 48 h of Bel treatment. Representative photographs are shown. Morphological effects induced by 0.5 and 2 μmol/L Bel after 48-h treatment, evaluated by phase contrast microscopy (magnification 100×) (**a**, **c**); evaluated by the acridine orange/ethidium bromide staining, shown by fluorescence microscopy (magnification 200×) (**b**, **d**). Markedly enlarged cells with bright yellow, condensed nuclei are visible. Evident reduction of cell density is observable

Microscopic observations were carried out to see if the diminished cell viability of LN-229 and LN-18 cells was accompanied by alterations in cell morphology and increased cell death. Observations showed that Bel-stimulated cells had different cell shape and cell density (Fig. 3). Figures 3a and c present the overview of LN-229 and LN-18 cells observed under phase contrast microscope after 48 h of incubation. The densities of both cell lines were visibly reduced following 48 h of treatment with Bel from a starting concentration of 0.5 μmol/L. The most significant suppression of cell proliferation was observed in cultures treated with 2 μmol/L of Bel. After 24 h of treatment, Bel showed minor effects on cell morphology and density (data not shown). In order to identify cells undergoing apoptosis, the AO/EtB staining was carried out and cells were viewed under a fluorescent microscope. This approach enables viable cells to be distinguished from dead ones by showing green-stained nuclei. In contrast, red-stained nuclei appear in apoptotic cells. Figures 3b and d demonstrate the representative photographs of AO/EtB-stained LN-229 and LN-18 cells exposed to 0.5 or 2 μmol/L of Bel for 48 h in comparison to the non-treated controls. Significant changes were observed in cells treated with 2 μmol/L concentration of Bel. These cells displayed an enlarged or swollen cell morphology together with increased cytoplasm as well as more condensed nuclei in comparison to controls. Additionally, LN-229 cells displayed a partial loss of the characteristic spindle-shaped morphology (Fig. 3b). In contrast, LN-18 cells demonstrated atypical jagged-like cytoplasm (Fig. 3d). Supplementation with Bel resulted in visible alterations of cell morphology and density, but there was a low proportion of cells observed with red-stained nuclei indicative of late apoptosis. This suggests that apoptotic events may occur in early stages, and are possibly reversible after removal of the stimulant.

### The effect of bel on apoptosis

**Fig. 4:**
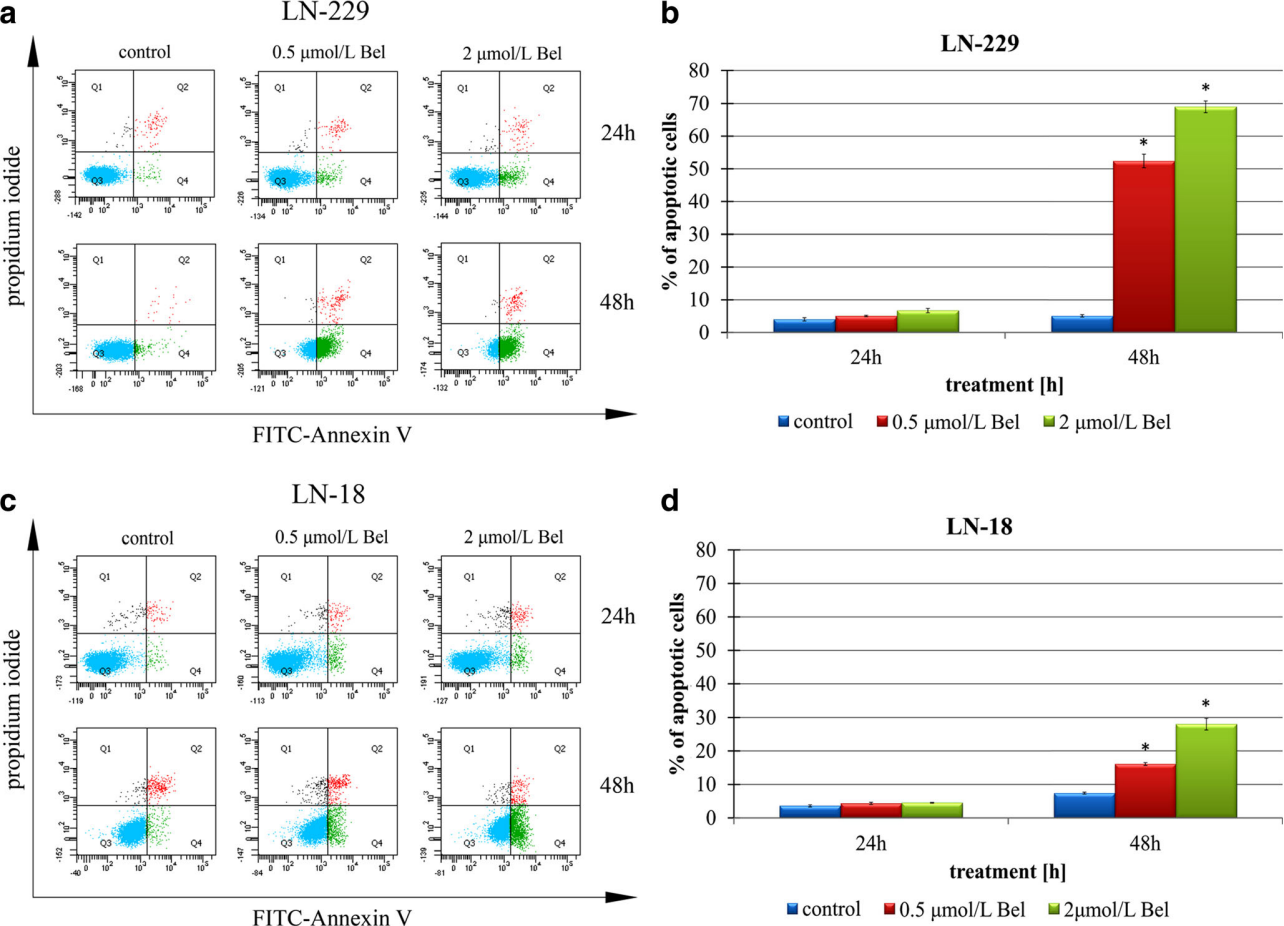
The effect of belinostat on apoptosis of glioblastoma cells. The cells were incubated with 0.5 and 2 μmol/L Bel for 24 and 48 h. Representative FACS analysis images for LN-229 (**a**) and LN-18 (**c**) cells subjected to Annexin V-FITC/propidium iodide staining are shown. Bar graphs presenting the percentage of apoptotic LN-229 (**b**) and LN-18 (**d**) cells, are demonstrated. Mean values from three independent experiments ± SD are shown. Significant alterations are expressed relative to controls and marked with asterisks. Statistical significance was considered if * *p* < 0.05

To further investigate the mechanism of the cytostatic effect of Bel in glioblastoma cells, we characterized the correlation between Bel and apoptotic cell death. In order to confirm the results of AO/EtB staining, both LN-229 and LN-18 cells were treated with Bel at the aforementioned concentrations. Cells undergoing apoptosis after treatment with Bel were assayed using flow cytometry analysis. The percentage of dead cells in cultures treated with 0.5 or 2 μmol/L of Bel for 24 and 48 h is reflected in Fig. 4. There was little effect of Bel treatment on the apoptosis of LN-229 and LN-18 cells incubated for 24 h. In contrast, a pronounced percentage of cells undergoing apoptosis was observed in LN-229 cells when treated with both 0.5 μmol/L Bel (52,4 % ± 2.06 %) and 2 μmol/L Bel (69 % ± 1.81 %; Fig. 4b) for 48 h. Interestingly, LN-18 seemed to be more apoptosis-resistant, as these values were only 16.1 % (± 0.32 %) and 28 % (± 1.74 %) for 0.5 and 2 μmol/L concentrations of Bel, respectively (Fig. 4d). It is noteworthy that the cells identified as apoptotic were mostly in the early phase of apoptosis. This might confirm the results of the microscopic observations where condensed but not red-stained nuclei were observed. This suggests the effects of Bel are be reversible after removal of the drug.

### The effect of bel on cell cycle distribution

**Fig. 5:**
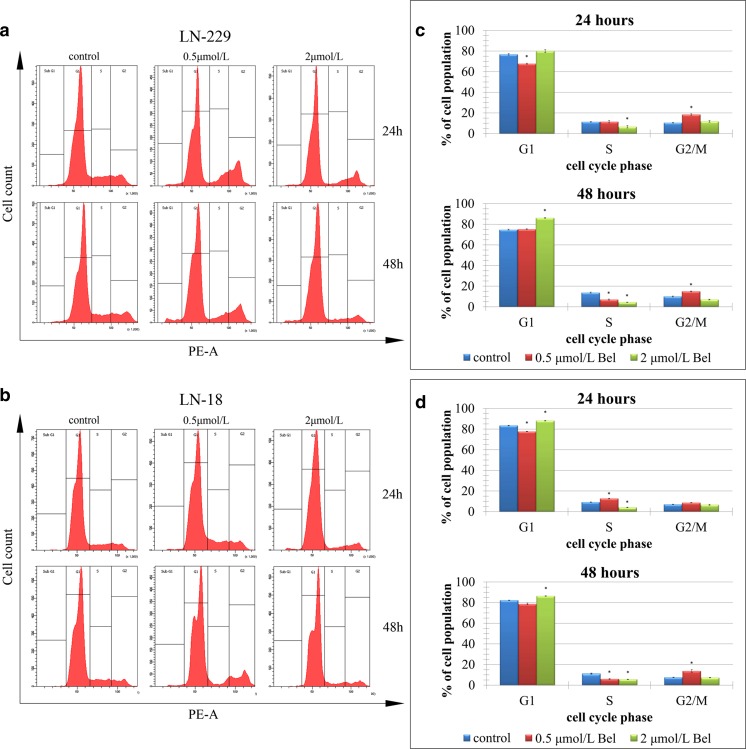
The effect of belinostat on cell cycle distribution of LN-229 and LN-18 glioblastoma cell lines. The cell cycle was measured by propidium iodide staining followed by flow cytometry analysis. Results are shown for cells treated with 0.5 and 2 μmol/L Bel for 24 and 48 h versus untreated controls. Graphical representation of the cell cycle profiles obtained from flow cytometry measurements in LN-229 (**a**) and LN-18 cells (**b**) are shown. Bar graphs presenting the percentage of cell cycle distribution in LN-229 cells after 24 h and 48 h (**c**); LN-18 cells after 24 h and 48 h (**d**) of treatment with belinostat. Significant alterations are expressed relative to controls and marked with asterisks. Statistical significance was considered if * *p* < 0.05

Flow cytometry was used to monitor the association between cell-cycle arrest and the anti-proliferative effect of Bel on LN-229 and LN-18. Bel treatment resulted in a slight interruption of the cell cycle distribution in both LN-229 and LN-18 cells (Fig. 5). Analysis of flow cytometry indicated that LN-229 as well as LN-18 cells cultured with Bel showed only modest differences in the distribution of cell cycle phases, with more pronounced effects observed after 48 h of incubation (Fig. 5a,b). Twenty-four hour treatment seemed to affect the cell cycle in both cell lines (Fig. 5c,d). After 48-h treatment of LN-229 cells with 0.5 μmol/L Bel there was a significant decrease in the S phase cells with a simultaneous increase in the number of cells in the G2/M phase in comparison to the control (Fig. 5c). A significant decrease in S phase cells and an increase in G1 phase cells was observed after treatment with 2 μmol/L Bel (Fig. 5c). Similar effects were noticeable in LN-18 cells after 48 h of culture with Bel (Fig. 5d). Both concentrations of Bel reduced the number of S phase cells (Fig. 5d). It can be concluded that Bel is incapable of inducing G1 or G2/M-dependent cell cycle arrest in LN-229 and LN-18 cells. However, it might slow down the progression of glioblastoma cells by decreasing the proportion of S phase cells and reducing the potential for proliferation.

### The effect of bel on GRP78 and GRP94 chaperone expression

**Fig. 6:**
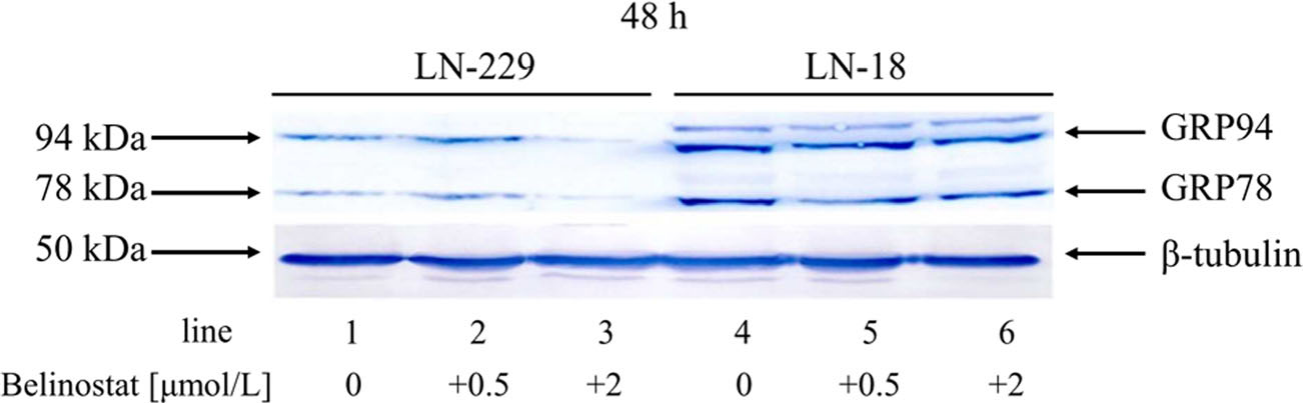
Expression of the ER chaperones in glioblastoma LN-229 and LN-18 cells. Western blot analysis of GRP78 and GRP94 expression in glioblastoma cells incubated with 0.5 and 2 μmol/L of belinostat for 48 h. Samples containing 20 μg of protein were submitted to electrophoresis and immunoblotting. A representative Western blot image is presented. β-tubulin was used as the loading control

Recent studies have demonstrated that HDAC inhibitors may interfere with the expression of molecular chaperones belonging to the heat shock protein 70 (HSP70) family. The ER-resident chaperone GRP78 is a member of this family of proteins. Recent research suggests GRP78 expression may also be affected by HDAC inhibition [11, 12]. It is well established that overexpression of ER chaperones, particularly GRP78, contribute to the enhanced proliferation and increased apoptosis resistance of glioma cells. This provides a promising target for the improvement of therapeutic responsiveness of these tumors. Given this research, we sought to determine how GRP78 responds to Bel treatment. GRP78 protein levels were examined by Western blot using anti-KDEL antibody. KDEL is a C-terminal amino acid signaling sequence (Lys-Asp-Glu-Leu) targeting proteins to the ER. Thus, an additional chaperone protein of 94 kDa, known as GRP94, is detected by this antibody. We observed that 48-h incubation with 2 μmol/L of Bel resulted in almost complete suppression of GRP78 and GRP94 expression in LN-229 cells, while it seemed to have almost no effect on the expression of these chaperones in LN-18 cells (Fig. 6). Interestingly, the basal level of GRP78 and GRP94, as seen in untreated control cells, was significantly different depending on the cell line. LN-18 cells appeared to express chaperone proteins more extensively than LN-229 cells (Fig. 6).

### The effect of bel on gene expression

**Fig. 7:**
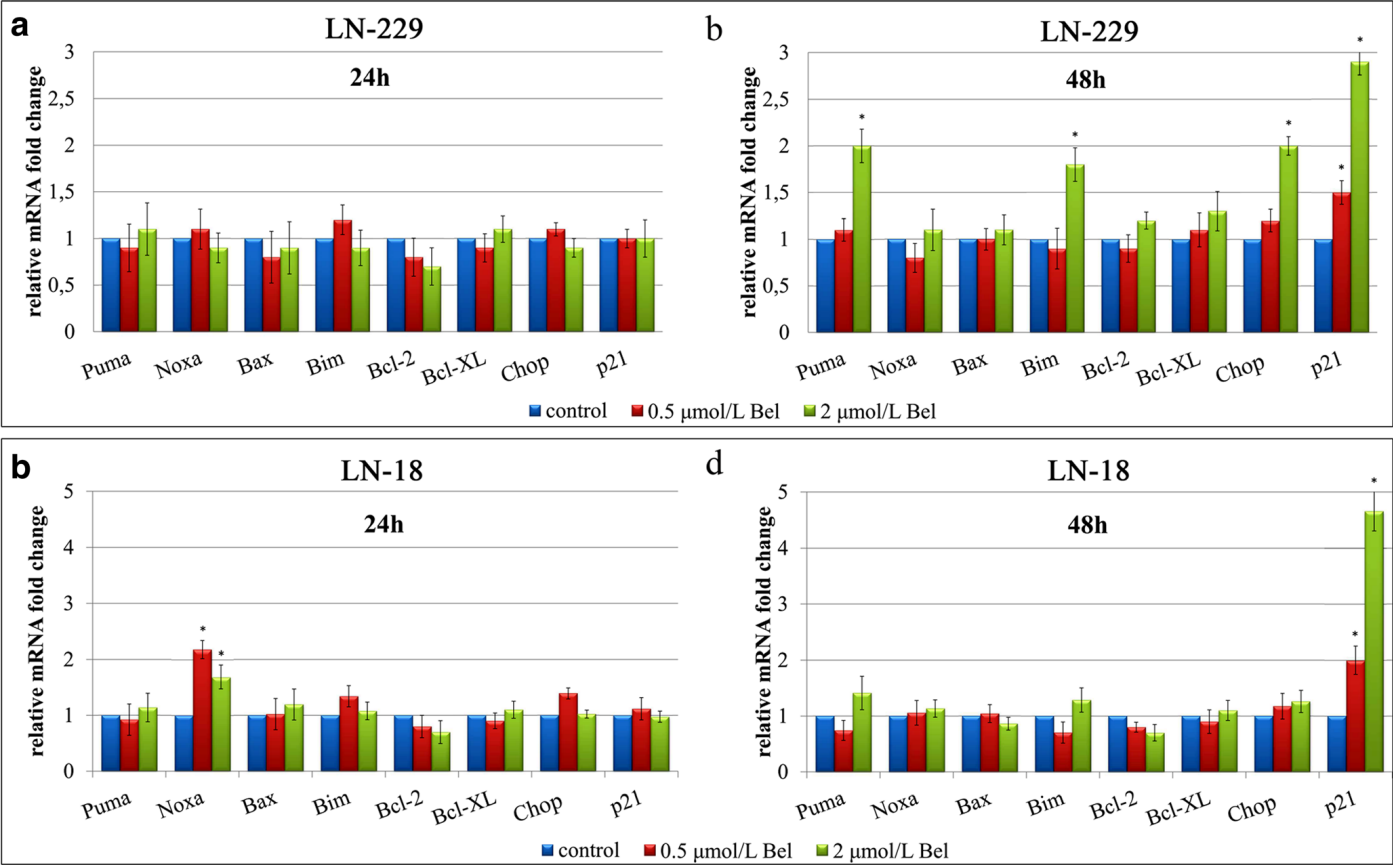
Relative quantification of gene expression in glioblastoma cells. Cells were treated with 0.5 and 2 μmol/L of Bel, and RNA was extracted from LN-229 cells cultured for 24 h (**a**) or 48 h (**b**); and LN-18 cells cultured for 24 h (**c**) or 48 h (**d**). Results are shown as a relative fold change in mRNA expression in comparison to untreated controls, where expression level was set as 1. Statistical significance was considered if * *p* < 0.05

The primary and most well-known mode of action of HDACIs is through the regulation of transcription and gene expression. RT-qPCR analyses of a set of genes involved in cell cycle control and apoptosis was carried out to determine if the apoptotic and cell cycle altering effects of Bel occurred due to the changes in gene expression (Fig. 7). The expression of pro-apoptotic genes: *Puma*, *Noxa*, *Bax*, *Bim*, *p21* and *Chop*, as well as anti-apoptotic *Bcl-2*/*Bcl-X*_*L*_ was analyzed to investigate this effect. Twenty-four hour treatment with 0.5 or 2 μmol/L of Bel resulted in no significant changes in the expression of these genes in both cell lines (Fig. 7a,c). These results suggest that 24 h incubation with Bel might be an insufficient amount of time to regulate gene expression patterns in glioblastoma cells. In contrast, 48 h treatment significantly altered transcription of some pro-apoptotic genes. The transcripts of *Puma*, *Bim* and *Chop* were significantly up-regulated in Bel-treated LN-229 cells (Fig. 7b). A 2-fold increase of *Chop* transcript in LN-229 was observed in cells treated with 2 μmol/L of Bel. No changes in *Chop* expression were found in LN-18 cells, regardless of the time of treatment or the dosage of the drug (Fig. 7b,d). These observations seem to be consistent with the results of GRP78 and GRP94 expression found in Western blot analysis, suggesting that alleviation of the expression of ER-related cytoprotective molecules might result in the simultaneous up-regulation of the pro-apoptotic factor *Chop* in LN-229 cells.

It is already established that Bel is an inducer of p21 expression. There were no changes observed in *p21* expression after 24 h of treatment (Fig. 7a,c). However, 48 h of incubation with Bel resulted in significant up-regulation of *p21* transcript in both cell lines (Fig. 7b,d). LN-18 cells tended to express *p21* to a higher extent than LN-229 cells. A 2-fold increase of *p21* expression in LN-18 cells incubated with 0.5 μmol/L Bel was observed, and an approximately 5-fold increase in cells cultured with 2 μmol/L of Bel was observed in comparison to the control (Fig. 7d). The corresponding values for LN-229 cells were 1.5 and 2.9, respectively (Fig. 7b).

## Discussion

Glioblastoma is a type of cancer that still poses a therapeutic challenge with poor overall prognosis. Surgery followed by adjuvant chemotherapy is still insufficient to achieve a long-term improvement in patients. Given this, new approaches aiming at inhibiting cancer cell growth remain an area of active research. Currently, it is widely recognized that HDACIs represent a promising class of anticancer agents with the capability to reverse aberrant epigenetic states observed in many malignancies. It is known that brain tissue expresses HDAC enzymes, thus the application of HDAC inhibitors as a potential antineoplastic agents in glioblastoma seems to be a logical approach to this issue [33].

Among various HDACIs, Bel shows potent anti-tumor activity against various types of cancer. Pre-clinical research has reported growth-inhibiting properties of Bel in pancreatic, prostate, and thyroid cancer cells [23–25]. Phase I and II clinical trials have demonstrated that Bel can be an efficient mono-therapeutic agent in cutaneous and peripheral T-cell lymphomas, as well as a co-therapeutic drug in combination with carboplatin and taxol in solid tumors [34]. Despite a considerable number of studies exploring the influence of Bel on various cancer cell lines, the molecular mechanisms initiated during Bel-mediated cellular responses are still poorly understood. More importantly, the effectiveness of Bel against glioblastoma cells has not been analyzed. In order to uncover more information about Bel activity in glioblastoma, we decided to investigate the effect of Bel treatment on LN-18 and LN-229 cell lines.

In our study, we observed that Bel is a potent inhibitor of HDAC activity in glioblastomas while achieving corresponding IC_50_ values in both LN-229 and LN-18 cell lines. Moreover, in line with the results of previous studies conducted on thyroid [25], colorectal [35], or pancreatic [24] carcinoma we found a prominent cytotoxic activity of Bel in both LN-229 and LN-18 cell lines, with the IC_50_ concentrations within the micromolar range. We also noticed that disturbances in cell viability were reflected in reduced cell proliferation and altered cell morphology. Microscopic observations confirmed that LN-18 as well as LN-229 cells subjected to Bel treatment demonstrated a visible decrease in cell density and changes in cell phenotype. We observed prominently enlarged cells with condensed bright yellow-stained nuclei. This is consistent with the results of previous studies demonstrating a phenotypic effect of other HDAC inhibitors such as phenylbutyrate, valproate, or vorinostat on glioblastoma cells [2, 10, 36]. Interestingly, no morphological differences between Bel-treated and control pancreatic tumor xenografts were found in the studies of Dovzhanskiy et al. [23]. This might suggest that glioblastoma cells may be sensitive to HDAC inhibitors in a specific way. However, further investigations are necessary to get a broader overview of the effects of Bel on cellular morphology.

Since nuclear condensation might be an indicator of cells undergoing apoptosis, we decided to analyze the anti-proliferative effect of Bel and determine if it can be explained by the induction of apoptotic cell death in LN-229 and LN-18 cells. Indeed, flow cytometry analysis confirmed the pro-apoptotic activity of Bel in both cell lines, showing mostly cells in the early-apoptotic stage. Forty-eight hour treatment resulted in remarkable apoptosis induction, while there was no increase in apoptotic cell death in neither LN-229 nor LN-18 cells cultured with Bel for 24 h (independently on the dosage). This seems to be in agreement with the results of other research demonstrating apoptosis to be one of the main mechanisms responsible for Bel-mediated cytostatic effect [23–25]. Despite this evidence, the molecular background underlying Bel-dependent apoptosis is not clear, and it seems that the effective targets of the drug may vary in a cell type-specific way. It has been reported that in Panc0327 and Panc1005 pancreatic cancer cells increased levels of cyclin-dependent kinase inhibitor p21 and death receptor 5 (DR5) were involved in apoptosis induction [23]. Another study reported reactive oxygen species (ROS) accumulation and interrupted RAS/RAF/ERK and PI3K/mTOR signaling pathways to be connected with apoptotic events in Bel-sensitive WRO82–1 and 8505C thyroid carcinoma cells [25]. Interestingly, in our study LN-18 cells were considerably more resistant to Bel-induced apoptosis, with less than half of the apoptosis rate observed for LN-229 cells. We decided to further investigate the main pro- and anti-apoptotic factors on the molecular level. It was observed that after 48-h treatment, Bel-stimulated LN-229 cells showed up-regulation of pro-apoptotic *Puma*, *Bim* and *Chop* transcripts. There were no changes observed in the expression of *Bax*, *Noxa*, nor *DR5* (data not shown). In contrast, in LN-18 cell levels in the presence of these pro-apoptotic mediators were unchanged. Our results are in partial agreement with the studies of Rao et al. who demonstrated elevated levels of pro-apoptotic mitochondria-connected proteins BIK, BIM, BAX, and BAK in breast cancer MCF7 cells after treatment with panobinostat, which is another pan-HDAC inhibitor [7]. This suggests that the deregulation of transcription of genes involved in mitochondria-mediated apoptosis might play a significant role in the regulation of Bel-dependent cell death in LN-229 cells. Furthermore, no significant alterations in the expression of anti-apoptotic factors *Bcl-2/Bcl-X*_*L*_ were observed in either cell line. This result seems to be in opposition to the observation made by Chien et al. who demonstrated that a decreased level of Bcl-X_L_ expression is associated with enhanced Bel-sensitivity of BxPc3 and AsPc1 pancreatic cancer cell lines [24]. This confirms that the molecular mechanisms initiated during Bel exposition might be highly tumor cell type-dependent.

Recently, the interaction between the ER stress-related UPR and histone deacetylase inhibitors has become a relevant research topic [7, 8, 11, 12]. In non-stressed cells, the molecular chaperone GRP78 is bound to its client proteins PERK (protein kinase-like ER kinase), ATF6 (activating transcription factor 6), and Ire1α (inositol-requiring enzyme 1α) anchored in the ER membrane, which prevents downstream signaling. It is established that UPR starts when the improperly folded proteins accumulate in the ER lumen. The GRP78 then dissociates from these three receptors to rescue the misfolded proteins. The GRP78-free sensors initiate a cascade of events aiming at saving the cell or to direct it into the apoptotic path when the stress reaches a threshold of severity. The pro-survival branch of the UPR is mainly associated with the enhanced activity of molecular chaperones such as GRP78 and GRP94, while the death tract is mostly CHOP-dependent [37]. This was associated with an elevated level of the pro-apoptotic transcription factor *Chop* in LN-229 cells, which may imply the involvement of the ER stress-dependent pathway in the process of apoptosis initiation in this cell line. This result is in line with the report of Rao et al. who also showed enhanced expression of CHOP in MCF7 cells after treatment with panobinostat [7]. However, in LN-18 cell line there were no signs of *Chop* up-regulation. Accordingly, Baumeister et al. also failed to demonstrate an increase in *Chop* expression after stimulation with trichostatin A, which is another well-established pan-HDAC inhibitor [38]. This suggests that there is no universal mechanism underlying HDAC inhibitors, and each agent may induce different molecular effects dependently on the cell type. Such discrepancies may propel researchers towards examination of new drugs and their specific effectiveness in various neoplastic entities.

It has been established that HDACIs may regulate the expression of GRP78 on a post-translational as well as transcriptional level [7, 11, 12, 38]. Elevated levels of GRP78 after stimulation with various HDAC inhibitors were already found in a set of cancer cell lines including glioblastoma, prostate, breast and colon cancer cells [7, 11, 12, 38]. Since GRP78 and GRP94 are believed to be the main anti-apoptotic mediators in ER-stressed cancer cells, an emerging role of these molecular chaperones has been associated with drug resistance in cancer. This research indicates that Bel deregulated the expression of glucose regulated proteins in glioblastoma cells. Western blot analysis showed that the expression of GRP78 as well as GRP94 was significantly down-regulated after 48-h treatment with 2 μmol/L of Bel in LN-229 cells. In contrast, protein levels of these chaperones seemed to be unchanged in LN-18 cells. Our hypothetical explanation for this phenomenon might be the fact, that LN-229 and LN-18 cells present different metabolic specificity correlated with differential gene expression patterns. Indeed, on the bases of metabolic profile analysis, Cuperlovic-Culf et al. have clustered LN-229 and LN-18 cells to distinct subtypes of glioblastomas [39]. In this respect, if Bel was undergoing slightly different metabolic dynamics in both cell lines, it might be possible to influence the acetylation process with regards to histone and non-histone protein acetylation. These results seem to be in line with the recent studies on glioblastomas showing that various subtypes of these tumors exist and most likely respond differently to treatments [40]. Nevertheless, the causes of this effect may also have a distinct molecular mechanism. This is likely the first report showing down-regulation of GRP78 by any of the known HDAC inhibitors. The ability to down-regulate GRP78 and GRP94 might be of special significance in context of glioblastoma treatment, where overexpression of these chaperone proteins is known to be correlated with enhanced chemoresistance [13, 14]. This UPR-modulatory effect may stimulate further research on potential co-treatments of Bel with other cytostatic agents targeting ER stress. This strategy has already been demonstrated to be effective in killing renal cancer cells [15, 41]. Preliminary results suggest synergistic killing of 769-P, 786-O and ACHN renal cancer cells by combined belinostat/bortezomib treatment [15]. Given this, application of proteasome inhibitors such as bortezomib, together with Bel in Bel-sensitive cells might require further research.

The mechanism of Bel-dependent cytostatic effect seems to be connected with the up-regulation of *p21* expression. The induction of this mechanism has already been demonstrated in various pancreatic, prostate, thyroid and bladder cancer cell lines subjected to Bel treatment [16, 23, 42]. In the present study, we also demonstrated the up-regulation of *p21* mRNA expression in both LN-229 and LN-18 cells after Bel treatment. The *p21* protein has been implicated in cell cycle inhibition, however a pro-apoptotic activity has also been suggested [43]. Indeed, Bel-induced *p21* overexpression has already been linked with G2/M cell cycle arrest in pancreatic and prostate cancer cells [22, 24]. Surprisingly, flow cytometry analysis failed to demonstrate clear G1 or G2/M cell cycle arrest, despite the up-regulation of *p21* expression. Significant deregulation of cell cycle in both cell lines was observed in the current study. This was mostly mirrored in the reduction of the S-phase cells, which may be indicative of a decrease in cells actively replicating their DNA, and thus contribute to perturbed cell proliferation.

Altogether, our findings demonstrate that Bel acts in a cell type-specific way and that mechanisms initiated during Bel-mediated responses are variable and complex. The results of our studies suggest a need for further analyses of Bel activity in animal models of glioblastoma. Performing tumor xenograft studies will bring the information about anti-glioblastoma effectiveness of Bel while considering its metabolic dynamics and efficiency in crossing blood-brain barrier. This would be an essential step into further understanding the anti-tumor potential of Bel in brain malignancies. Thus, it seems necessary to continue the in vivo research on Bel in brain tumors. Being able to define all possible signaling pathways initiated during Bel exposure might lead to the successful implementation of Bel as a potential mono-therapeutic drug or a co-therapeutic agent in glioblastoma therapy.
